# Towards a Reliable and Rapid Automated Grading System in Facial Palsy Patients: Facial Palsy Surgery Meets Computer Science

**DOI:** 10.3390/jcm11174998

**Published:** 2022-08-25

**Authors:** Leonard Knoedler, Helena Baecher, Martin Kauke-Navarro, Lukas Prantl, Hans-Günther Machens, Philipp Scheuermann, Christoph Palm, Raphael Baumann, Andreas Kehrer, Adriana C. Panayi, Samuel Knoedler

**Affiliations:** 1 Department of Plastic, Hand and Reconstructive Surgery, University Hospital Regensburg, 93053 Regensburg, Germany; 2 Department of Surgery, Division of Plastic Surgery, Yale School of Medicine, New Haven, CT 06510, USA; 3 Department of Plastic Surgery and Hand Surgery, Klinikum Rechts der Isar, Technical University of Munich, 81675 Munich, Germany; 4 Regensburg Medical Image Computing Lab, Ostbayrische Technische Hochschule Regensburg, 93053 Regensburg, Germany; 5 Department of Surgery, Division of Plastic Surgery, Brigham and Women’s Hospital, Harvard Medical School, Boston, MA 02115, USA

**Keywords:** Bell’s palsy, idiopathic facial paralysis, facial palsy, machine learning, grading systems, automated grading, artificial intelligence

## Abstract

Background: Reliable, time- and cost-effective, and clinician-friendly diagnostic tools are cornerstones in facial palsy (FP) patient management. Different automated FP grading systems have been developed but revealed persisting downsides such as insufficient accuracy and cost-intensive hardware. We aimed to overcome these barriers and programmed an automated grading system for FP patients utilizing the House and Brackmann scale (HBS). Methods: Image datasets of 86 patients seen at the Department of Plastic, Hand, and Reconstructive Surgery at the University Hospital Regensburg, Germany, between June 2017 and May 2021, were used to train the neural network and evaluate its accuracy. Nine facial poses per patient were analyzed by the algorithm. Results: The algorithm showed an accuracy of 100%. Oversampling did not result in altered outcomes, while the direct form displayed superior accuracy levels when compared to the modular classification form (n = 86; 100% vs. 99%). The Early Fusion technique was linked to improved accuracy outcomes in comparison to the Late Fusion and sequential method (n = 86; 100% vs. 96% vs. 97%). Conclusions: Our automated FP grading system combines high-level accuracy with cost- and time-effectiveness. Our algorithm may accelerate the grading process in FP patients and facilitate the FP surgeon’s workflow.

## 1. Introduction

As the most common cranial nerve disease, facial palsy (FP) has various aetiologies with idiopathic forms (Bell’s palsy; BP) accounting for 60–75% of cases [1,2,3]. FP shows an annual incidence rate of up to 40 cases per 100,000 population with equal rates in male and female patients [4,5,6]. The mean age of onset ranges from 45–56 years of age [7,8]. Depending on the lesion localization, FP can be caused by trauma, or following viral or bacterial infections (e.g., HSV-1, VZV, Lyme disease), neoplasms, or surgery [9,10]. Additionally, autoimmune diseases, such as Sjögren- or Guillan–Barre syndrome, are associated with FP [11]. Yet, the exact etiology in acute FP cases remains the subject of ongoing research [12]. Based on the complex course of the facial nerve and the diverse quality of fibers, FP patients may present with a plethora of symptoms including disfiguring facial asymmetry, involuntary mimic movements, insufficient mouth and lip tonus, as well as inappropriate emotional expression [10,13]. Further, incomplete eyelid closure leads to the pathognomonic Bell’s phenomenon (i.e., visible upward and outward movement of the eye during eye closure) [14]. Whereas loss of the stapedius muscle is associated with hyperacusis, impairment of the visceral and sensory function of the facial nerve and the stapedius muscle reduces saliva flow, causes dry eye disease, and results in atypical taste sensations [5,15]. Besides these physical symptoms, which can even extend to exposure keratopathy and vision loss, FP patients suffer from social withdrawal and physiological stress and report decreased quality of life [15,16,17]. Despite complete remission in 70–80% of FP patients within the first year after onset, FP symptoms can persist in varying severity levels and coincide with muscular hypo- and hyperactivity, synkinesis (i.e., involuntary muscle contractions), or postparalytic facial nerve syndromes [5,9,18].

To trace the time course of FP disease more accurately, FP practitioners differentiate acute, subacute, and chronic phases of FP [19]. FP guidelines commonly outline the key role of rapid and reliable diagnosis and treatment decisions for successful disease control [20]. However, the FP diagnosis is one by exclusion, requiring the investigation of potential risk factors and eventual medical history of previous FP, but also includes clinical neurological investigation, lumbar puncture, and blood chemical examination, as well as diagnostic imaging, such as X-ray or MRI examination [12]. To categorize the level of nerve damage and facial dysfunction, electroneuromyography and different clinical as well as computer-aided scoring systems, such as eFACE, can be utilized [21,22,23]. Such classification frameworks are valuable tools in the initial examination of FP patients and in ensuing treatment [11,23]. The six-point House and Brackmann scale (HBS) comprises scores from I (i.e., physiological facial movements) to VI (i.e., complete paralysis) and, since its introduction in 1985, has been the most commonly applied FP grading system [24]. Over the last three decades, more advanced classification systems have enlarged the FP examiner’s diagnostic repertoire, namely the Sunnybrook facial grading system, the eFACE system, and the Emotrics platform [25,26,27]. Implementing more detailed clinical parameters, the Sunnybrook facial grading system combines robust reliability and high-level sensitivity [28,29]. Whereas previously mentioned grading systems are limited by their subjectivity, novel computer-aided assessment tools using machine learning algorithms for quick and accurate localization of facial landmarks constitute a state-of-the-art option for objective FP measurements given their high-throughput capacity and digital availability [23]. 

FP therapy is multimodally conceptualized [30,31,32]. The conservative treatment landscape for FP ranges from immunosuppressive drug regimens to alternative complementary therapies, such as acupuncture and physical therapy [33]. Randomized controlled trials have confirmed the beneficial use of oral corticosteroids in acute FP, yet the clinical effects of antiviral medication and insulin-like-growth-factor-1, as well as surgical decompression of the facial nerve, are contentiously discussed [34,35,36,37]. By weakening the overactive face-side to target synkinesis and facial imbalances, Azizzadeh et al., as well as Labbe et al., introduced novel surgical techniques, namely selective modified neurectomy and myectomy [38]. The most common mimic muscles treated with myectomy are the Depressor labii inferioris and Depressor anguli oris muscles, which hinder a full-effort patient smile. The marginal mandibular branch of the facial nerve represents the most frequently addressed neural structure in selectively modified neurectomy [39,40]. Dynamic rehabilitation is considered the current gold standard in facial nerve rehabilitation including neurotization procedures, such as direct nerve regeneration, cranial nerve transfer (e.g., masseteric-to-facial nerve transposition), and cross-face nerve grafts [41,42,43]. Moreover, patients with long-term (i.e., ≥18 months after onset) uni- and bilateral facial paralysis may undergo free and regional (microsurgical) muscle transfer. In particular, masseter nerve-innervated gracilis muscle transfer yields promising functional and aesthetic outcomes by imitating the function of the Zygomaticus major muscle [44,45]. 

There is no all-embracing guideline, so treatment decisions remain an individual case-to- case process, in which clinicians have to meticulously assess surgical or medicinal risks and weigh up aesthetic ideals and functional requirements. Thus, reliable diagnosis and disease classification are mandatory for effective and substantial treatment selection. Given the increased necessity for time- and resource-effective clinical workflows, prompt and reliable grading of FP disease is imperative. Computer-aided FP grading systems are set to become clinical routine in FP scoring. Therefore, we aimed to develop an easy-to-use, rapid, and highly reliable automated machine learning algorithm to classify images of FP patients according to the HBS.

## 2. Materials and Methods

Image datasets of 86 patients seen at the Department of Plastic, Hand, and Reconstructive Surgery at the University Hospital Regensburg, Germany, between June 2017 and May 2021 were used to train the neural network. Each case comprised nine images; these were frontal images of the following nine poses: (1) Face in repose; (2) raising the eyebrows; (3) smile with mouth closed; (4) full-denture smile; (5) pursing the lips; (6) gentle eye-closure; (7) forced eye-closure; (8) wrinkling the nose; and (9) depressing the lower lip. 

The first pose focused on the facial symmetry at rest, while the second image depicted the forehead to capture the function and movement of the muscles around the forehead area (i.e., intentional wrinkling). Eyelid closure was captured statically, and insufficient eye closure was recognized when the sclera was visible. The facial expressions in the remaining images showed the mouth in different positions to visualize asymmetries between the two sides of the face. 

The images were divided into two non-overlapping datasets (i.e., training set and validation set) used either for training the neural networks or evaluating their performance. Given that classes I, II, III, and V of the HBS were underrepresented in our study population, the corresponding images were inserted multiple times into the training data (i.e., oversampling) to resolve this imbalance and yield more refined outcomes. The validation set remained unaltered. 

The modular form and the direct form were used to test two different classification approaches. In the modular form, the different factors of the HBS were considered as single modules, which are expert systems, each optimized to focus its classification efforts on one of four key features: Face symmetry, eyelid closure, mouth, and forehead. To improve the neural network’s performance, (i) the data were pre-processed by first removing irrelevant image sections (e.g., background, neck, and torso) and (ii) then using marker points to divide the nine images into subregions that (iii) finally served as input to the modules. 

By using an automaton (i.e., a set of rules) or the row sum of the predicted probabilities per module for each degree of HBS (e.g., symmetry score for HBS I + forehead score for HBS I + eyelid closure score for HBS I + mouth score for HBS I = row sum for HBS I), the individual predictions of the four facial modules were combined to predict the HBS value as output. The direct form assigned an HBS value ranging from I to VI directly to the FP. 

**Figure 1:**
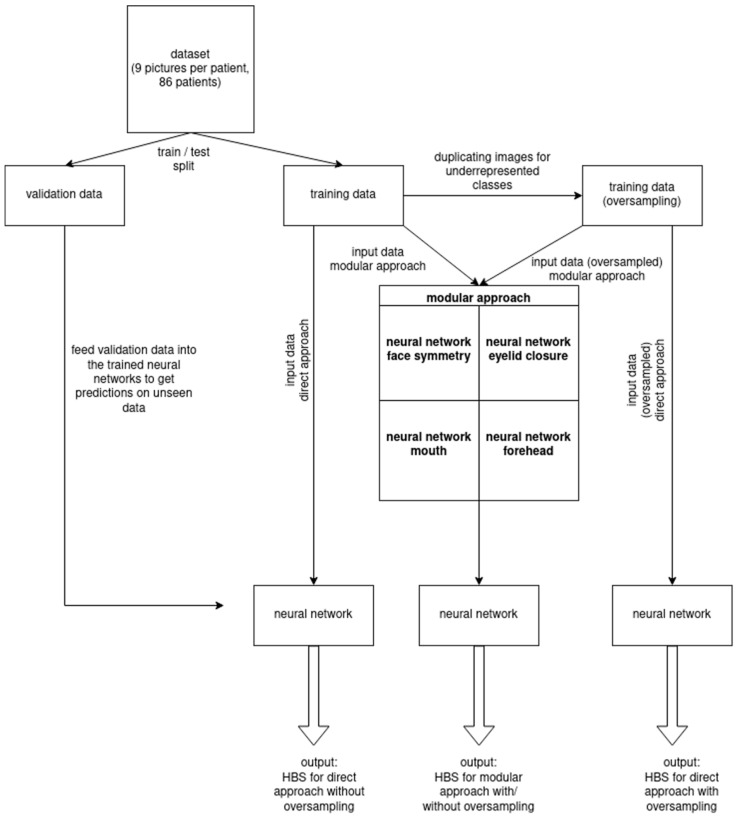
Schematic workflow including neural network training and validation process.

Different procedures were utilized to process the patient image series including the nine aforementioned photos. In the sequential method, the nine patient images were entered stepwise into the neural networks. By additionally applying Early Fusion means, the images were joined in advance and inserted into the neural networks as one package. When performing Late Fusion, each image was assigned its own network for each module. The evaluation of results was performed based on precision and recall criteria, which were calculated to obtain the F1-score (i.e., the harmonic mean of precision and recall). Precision describes the fraction of true positive samples among the samples that the model classified as positive, while recall/sensitivity is the fraction of samples classified as positive among the total number of positive samples. Utilizing the Phyton—and Fsolve—program, approximative values of true positive, true negative, false positive, and false negative were calculated in order to determine accuracy rates. Rebuilding and comparison of F1-scores were performed for control purposes showing deviation values of 0.5–1.5%. The entire workflow is illustrated in Figure 1.

## 3. Results

### 3.1. Direct Classification Approach Yielded Significantly Enhanced Outcomes

The direct classification form yielded significantly superior results compared to the modular form in 100% (n = 86) of cases. Further details are summarized in Table 1.

### 3.2. Early Fusion Showed Refined Results

In comparison to the Late Fusion technique or the sequential method, Early Fusion yielded significantly refined results (F1-scores: Early Fusion = 1.000 vs. Late Fusion = 0.927 vs. sequential method = 0.914; accuracy scores: Early Fusion = 1.000 vs. Late Fusion = 0.963 vs. sequential method = 0.968) across all samples. An in-depth outcome comparison is provided in Table 2.

### 3.3. Oversampling Technique Did Not Influence Classification Performance of the Neural Networks 

Using the oversampling technique did not significantly enhance the classification performance, as the maximal positive differences in F1-scores and accuracy achieved by oversampling were +0.032 (direct form vs. Late Fusion: 0.895 vs. 0.927) and +0.026 (direct form vs. sequential method: 0.968 vs. 0.942) (Table 2). The maximum negative differences in F1-scores were −0.025 (module form vs. sequential method: 0.355 vs. 0.330) and in accuracy were −0.021 (module form vs. sequential method: 0.621 vs. 0.600).

### 3.4. Combination of Early Fusion and Direct Form Yielded Optimized Classification Scores 

F1 and accuracy scores of 1.000 were achieved by combining the direct form with Early Fusion. In this case, all samples of the validation set were classified correctly, regardless of whether oversampling was used or not.

### 3.5. External Databases and Recently Used- (LRU-) Caches Accelerated Runtimes

Processing time was 151 ms for all nine images per patient using external databases and increased LRU-caches up to 55.

### 3.6. Direct Classification Approach Yielded Enhanced Outcomes

The direct classification form yielded significantly superior results compared to the modular form in 100% (n = 86) of cases. Further details are summarized in Table 1.

### 3.7. System Evaluation with F1-Score and Accuracy

Variables based on recall and precision criteria have been used to evaluate our grading system in order to make the introduced tool comparable to other state-of-the-art automated grading algorithms. The original patient data (n = 86) were subdivided into training (75%; n = 65) and validation (25%; n = 21) sets, resulting in two different cohorts. Approximative values of true positive (TP), true negative (TN), false positive (FP), and false negative (FN) results were calculated by analyzing the validation set after training our algorithm with the training dataset. The accuracy and sensitivity of the classification system are determined by the accuracy score and the F1-Score. Both were calculated for different processing methods (i.e., sequential, Early fusion, Late fusion) in combination with or without oversampling. The F1-Score is defined by the harmonic mean of precision and recall. Recall measures the extent of errors caused by FN, whereas precision measures the error caused by FP. The proximity of measurement results to the true HBS grading value is described as the accuracy, which was calculated as the proportion of TP and TN in all evaluated cases. 

## 4. Discussion

Over the past decade, technical applications on handheld devices have developed into an integral part of the clinical workflow across different specialties. Such applications range from weight-loss support and the management of Diabetes-Mellitus Type II and hypertension, to app-based diagnosis protocols [46,47,48,49]. Particularly in FP patients, timely and reliable diagnosis is crucial for effective therapy and positive patient outcomes. Prompt decision-making has been shown to limit permanent sequelae including axonal loss and muscle atrophy [50,51]. The first 72 h after onset represent the most crucial timeframe in FP therapy including the diagnostic option of intraoperatively stimulation of the distal facial nerve segments, as well as the beneficial prescription of corticosteroids [21,52]. Even in this early disease stage, ocular dryness and insufficient eye closure (i.e., lagophthalmos) can induce corneal microlesions finally resulting in keratitis, corneal ulceration, and permanent vision loss [53,54,55]. Here, the implementation of automated FP grading algorithms in the clinical workflow, as well as in home-diagnosis for laypersons, can increase time-efficient disease evaluation in order to ensure timely treatment initiation [56]. Our machine learning (ML) algorithm may also address the persisting undertreatment of FP patients who face waiting times of several months and insufficient referral to specialized care—only 7% of patients are reported to be referred to specialized care [57]. Utilizing our approach as a first-gate strategy, the necessity for timely evaluation by FP specialists can be better gauged, limiting medical resource wastage.

The proposed ML algorithm is compatible with both thick client systems (i.e., a networked computer system with most resources installed locally) and thin clients (i.e., a networked computer system with most resources distributed over a network), which offers the advantages of low maintenance costs, simple usage, and widely available access on Android and IOS operating systems. Consequently, a mobile-based application of our approach can be realized without complex and expensive hardware, in contrast to standard clinical intervention systems [58]. Each measurement tool required, such as the camera and processor, is already included in a conventional mobile phone, so the possibility of visual self-diagnosis of laypersons and clinicians with minimal experience in FP treatment/diagnosis comes into clinical reach [59]. Moreover, the omnipresence of various mobile devices, as well as our highly accurate algorithm free of development costs, make the automated grading system a promising low-budget and easy-to-use application.

The HBS is still the most commonly used clinical grading scale for FP patients in the US [60]. Yet, the emergence of other classification systems indicates the need for more (technologically) advanced platforms [61]. Our algorithm builds upon the clinically established HBS and automatizes the workflow toward a high-throughput system yielding reliable outcomes. Further, we overcome the ongoing challenge of subjective clinical FP scoring, caused by variedly trained or skilled clinicians and by a broad scope for interpretation. Our objective automated assessment tool may allow for clinician staff release, reduction in misdiagnoses, interindividual comparability, and consistency in FP scoring [62,63]. Indeed, an app-based version of our algorithm would be the first of its kind in the clinical classification process of FP [49,64]. In contrast to maintenance and cost-intensive measurement tools for clinical diagnosis, laser speckle contrast imaging (LSCI) and detailed 3-dimensional recording RGB-D cameras, let alone 3D-based techniques, we aimed for cost-efficiency, as well as layperson-friendly usability for patients at home, private practices, and clinics [57,64,65]. Consequently, efficient FP monitoring, as well as the prevention of recurrent FP, in- and outside of clinical institutions and private practice can be realized. 

The outputs of neural network measurements showed further benefits versus algorithms that recognize action units (AU) qualified by the Facial Action Coding System (FACS) [66]. Such systems require tedious recording procedures. For example, the 1992-developed OSCAR-system necessitates a 20-min video recording for FP assessment [67,68]. Furthermore, in 1999, Frey et al. introduced a computer-aided system for FP assessment that requires analysis durations of more than three hours per patient (including manually patient marking, video recording and processing, and data analysis with Facialis software), and the results have been tracked with an accuracy of nearly 99% [69]. In contrast, the proposed approach allows for saving single images in a cache to achieve real-time computation in outputs, as well as time-efficient diagnosis options in the hectic clinical routine. We can, therefore, ensure decreased computational costs due to the implementation of external databases or last recently used- (LRU-) caches [70]. The processing time of 151 ms is comparable to the processing time reported by Haase et al. (108 ms) who used low-dimensional AAM-parameters as features instead of high-dimensional descriptions [66]. Alternative evaluation concepts, such as the Facegram 3D and the mentioned 3-Dimensional Video System by Frey et al., perform FP assessment via clinician-marked anatomical landmarks [65,69]. Yet, utilizing anatomical landmarks for the assessment of facial functions entails the risk of subjective and random landmark positioning. With our marker-free evaluation system, we reduce inter- and intraobserver variability and promote reproduceable results [71]. The present automated scoring system is trained to recognize the accurate FP degree scoring only nine images, which benefits user-friendly performance and suitability for a routine examination, taking into consideration that it is much easier (and faster) to take nine standardized photographs than reproducible videos. 

Compared to previously proposed automated FP grading systems, our algorithm yields an optimal accuracy of 100% in the early fusion mode. Previous research work on Active Appearance Models (AAMs) reported accuracy levels of 88% and up to 94% utilizing Multiresolution Local Binary Patterns (MLBP) [71,72]. The present algorithm also outperformed state-of-the-art interventions, such as the concept proposed by Azuma et al. yielding accuracy values of 97% [64,70]. A 2018 study featuring convolutional neural networks (CNNs) calculated accuracy values between 89 and 96%, depending on the identification of different FP severity degrees [73].

There are three main prerequisites in FP evaluation: (1) Time-efficiency, (2) robust inter- and intraobserver reliability, and (3) user-friendly application. When combining these attributes, the implementation of an application based on the proposed ML algorithm can easily be translated into a clinical reality. Further, the algorithm can be directly integrated into broader mobile Health (mHealth) applications, which associate FP classification as part of a universal examination protocol gathering information on various clinically relevant parameters (e.g., sleep tracking, nutritional scores, and psychological wellbeing). This dataset may provide a comprehensive picture of the patient’s status and allow for more refined therapy decisions. In this project, we aimed for a novel method of ML-based FP classification accumulating all relevant benefits of a real-world automated examination tool. 

**Figure 2:**
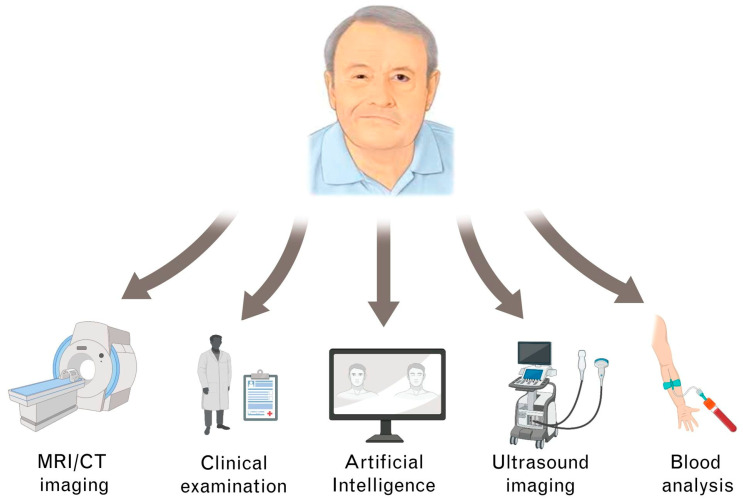
Different diagnostic pathways in facial palsy (FP) management.

In summary, our algorithm may enlarge the FP surgeon’s diagnostic arsenal commonly consisting of clinical examination, blood tests, and diagnostic imaging [11,74,75] (Figure 2). In the preoperative setting, our algorithm may help to reduce overall waiting times for FP patients by accelerating the FP surgeon’s workflow. In the senior author’s experience, thorough grading of FP patients based on the HBS can take up to five minutes or even longer in complex FP patient subsets (e.g., neurofibromatosis patients). It is not unusual for FP specialists to examine more than 30 FP patients per day. The algorithm could sufficiently perform this task. Given the structured simplicity of our model, the classification process could be assigned to technical assistants, saving the FP surgeon even more work time. This way, FP surgeons could dedicate more time toward individualized patient information. Intraoperatively, the algorithm may enable direct objective measurements. Based on these measurements, FP surgeons could, for example, readjust the placement of free muscle transplants before final wound closure to ensure feasible postoperative outcomes. In the postoperative follow-up, our algorithm could provide objective and intercomparable evaluations (theoretically even in the hand of FP patients). For interdisciplinary treatment in FP therapy, the objective classifications of our algorithm could serve as a common ground and facilitate joint therapeutic efforts.

## 5. Limitations

The results of this study ought to be interpreted in light of the following limitations. In our patient population, severe degrees of FP diseases were more prevalent than minor FP cases. However, we included the most common typical scenarios to generate a representative patient cohort and performed oversampling to balance the neural network input. Furthermore, our patient sample was limited to 86 individuals with FP. While this sample size allows for reliable proof of principle, further larger-scale studies would elucidate the strengths and limitations of the present algorithm. Over the four years of data collection, the authors have made consistent efforts toward uniform photographic documentation. New cameras for patient image documentation were purchased in 2021. Yet, it is unlikely that the marginal differences in the high-quality patient images of different cameras may have influenced the algorithm output since each patient image was computed using standardized pixel sizes (e.g., 640 × 300 for the oral region). In comparison to other models we developed for the purpose of automated grading, we anecdotally found the algorithm of the present study to outperform the other models. Yet, such performance differences remain to be corroborated in larger-scale studies.

## 6. Conclusions

We present an ML-based and easy-to-use evaluation tool for FP with high classification accuracy and rapid automated grading of FP images. This combination is a promising step toward optimized FP diagnosis.

## Figures and Tables

**Table 1:** F1-scores compared by classification approach without oversampling.

(A)	Processing Method	Oversampling	F1-Score	Accuracy
Module form	sequential	no	0.355	0.621
		yes	0.330	0.600
	Early Fusion	no	0.980	0.990
		yes	0.967	0.983
	Late Fusion	no	0.817	0.900
		yes	0.808	0.895

**Table 2:** F1-scores for module form and direct form for different processing methods, with and without oversampling for classification on validation set.

(B)	Processing Method	Oversampling	F1-Score	Accuracy
Direct form	sequential	no	0.884	0.942
		yes	0.914	0.968
	Early Fusion	no	1.000	1.000
		yes	1.000	1.000
	Late Fusion	no	0.895	0.964
		yes	0.927	0.963

## Data Availability

Not applicable.
